# High-throughput phenotyping of buckwheat (*Fagopyrum esculentum* Moench.) genotypes under water stress: exploring drought resistance for sustainable agriculture

**DOI:** 10.1186/s12870-025-06429-6

**Published:** 2025-04-08

**Authors:** Michal Antala, Marek Kovar, Lucia Sporinová, Andrej Filacek, Radosław Juszczak, Marek Zivcak, Aida Shomali, Raghavendra Prasad, Marian Brestic, Anshu Rastogi

**Affiliations:** 1 Laboratory of Bioclimatology, Department of Ecology and Environmental Protection, Faculty of Environmental Engineering and Mechanical Engineering, Poznan University of Life Sciences, Piątkowska 94, Poznań, 60-649 Poland; 2 Department of Plant Physiology, Faculty of Agrobiology and Food Resources, Slovak University of Agriculture, A. Hlinku 2, Nitra, 949 76 Slovak Republic; 3 Department of Botany and Genetics, Faculty of Natural Sciences and Informatics, Constantine the Philosopher University in Nitra, Nábrežie mládeže 573, Nitra, 949 01 Slovak Republic; 4 Department of Environmental Horticulture, Royal Horticultural Society, London, GU23 6QB UK; 5 Department of Botany and Plant Physiology, Faculty of Agrobiology, Food, and Natural Resources, Czech University of Life Sciences Prague, Kamycka 129, Prague, 165 00 Czech Republic

**Keywords:** Adaption strategies, CO_2_ assimilation, Genetic diversity, Phenotyping, Drought

## Abstract

**Background:**

As global agriculture faces the challenge of climate change, characterized by longer and more severe drought episodes, there is an increasing need for crop diversification and improved plant breeding. Buckwheat is one of the climate-resilient candidates for future important crops with remarkable adaptability to various biotic and abiotic stresses. As an underbred crop, a large number of genotypes should be assessed for the breeding of superior plants. Therefore, this study investigates the response of various buckwheat genotypes to water stress by high-throughput phenotyping and auxiliary plant physiology measurements.

**Results:**

We assessed six buckwheat genotypes from different regions under mild and severe water stress, focusing on morphological and physiological changes to understand drought tolerance mechanisms. Our findings revealed that reallocation of assimilated carbon from growth to secondary metabolite production is a common response to drought stress. Among the genotypes tested, Panda emerged as the most drought-resistant, with its morphology remaining the most stable under mild water stress and its ability to rapidly accumulate protective pigments in response to drought. Silver Hull also demonstrated resilience, maintaining its aboveground biomass under mild water stress at levels comparable to the control group. Additionally, the response magnitude to drought stress was linked to the biomass production potential of the genotypes, which was higher for those from warmer regions (Bhutan, Zimbabwe) and lower for those from colder regions (Poland, Canada).

**Conclusion:**

The diversity in genotypic responses highlights the significant role of genetic variability in shaping drought resistance strategies in buckwheat. This research not only enhances our understanding of buckwheat’s physiological responses to water stress but also holds promise for developing drought-resistant buckwheat varieties. These advancements are crucial for promoting sustainable agriculture in the face of climate change.

**Supplementary Information:**

The online version contains supplementary material available at 10.1186/s12870-025-06429-6.

## Introduction

In today’s world, a significant portion of farmland is dedicated to the cultivation of profitable monocultures, such as wheat and corn. However, the growing recognition of minor and pseudo-cereal crops offers a promising alternative, as they require lower inputs and often produce specific nutrients that surpass major cereal crops. Adopting such crops not only enhances biodiversity but also promotes environmental sustainability. Recent research has revealed that minor and pseudo-cereal crops, including buckwheat, exhibit remarkable adaptability to various biotic and abiotic stresses, positioning them as climate-resilient candidates [1, 2].

Buckwheat stands out as an essential functional food, distinguished by its high levels of essential amino acids, vitamins, and phenolic compounds. Its nutritional properties, antioxidant capacity, and suitability for preparing gluten-free products have elevated its significance in Europe. Milled dried plants were even suggested as natural colorants for pasta and other food items [3]. Moreover, scientific literature has reported numerous health benefits associated with buckwheat consumption, such as reduction of hyperlipidemia, blood pressure regulation, and potentially positive effects on patients with diabetes or Alzheimer’s disease [4–6].

Given the escalating impact of climate change, rising temperatures, more pronounced and more often temperature extremes, altered precipitation pattern and the intensification of droughts poses a significant threat to global crop production and food security [7, 8]. By inducing stomatal closure, drought stress hinders vegetative growth, reduces photosynthesis, and disrupts reproduction, resulting in yield reduction [9]. Furthermore, drought stress leads to the generation of reactive oxygen species (ROSs) that are harmful to plant cells and impair photosynthetic machinery [10]. However, plants have developed strategies to combat drought, mainly by reducing water loss to maintain sufficient photosynthesis and production of protective secondary metabolites to avoid the detrimental effect of ROS [11]. Nonetheless, the tolerance mechanisms depend on the severity and duration of stress [12]. In this context, buckwheat has demonstrated high resistance to various environmental constraints, making it an intriguing subject for drought stress research [9]. Despite its resilience, there is limited information available about buckwheat’s response to drought stress and the physiological traits associated with drought tolerance.

Conventional breeding methods often face challenges due to the inability to efficiently evaluate single plants, leading to prolonged breeding periods to achieve desired characteristics [13]. In contrast, rapid and precise phenotypic assessment through plant phenotyping offers a powerful alternative. Phenotyping not only facilitates the identification of elite plants but also enables the functional analysis of specific traits crucial for crop improvement. High-throughput phenotyping has become an essential research tool, particularly in breeding programs, allowing breeders to develop buckwheat cultivars with higher adaptability to diverse environmental conditions [14].

In light of these considerations, this study attempts to utilize a plant phenotyping approach for exploring the dynamics of water stress responses in different buckwheat genotypes originating from various continents (Africa, Asia, North America, and Europe). Because of different climatic conditions in areas of genotypes’ origin, we hypothesize that they will exhibit divergent responses to drought stress with the genotypes from generally warmer regions, such as Africa, being the most drought resistant. This research aims to unravel the mechanisms underlying buckwheat’s drought tolerance by investigating the physiological and morphological changes in response to water stress. Ultimately, these findings may pave the way for developing climate-resilient buckwheat cultivars, contributing to sustainable agriculture practices and environmental conservation in the face of an ever-changing climate.

## Materials and methods

### Plant material and growth conditions

Plants of six common buckwheat genotypes of different provenience were used in the experiment. The genotypes were chosen to represent different geographic regions of the world to capture the genetic variability with the focus on different local climatic conditions. The genotypes used were PI 482,597 from Zimbabwe (Southern Africa; “Zimbabwe”), PI 481,640 from Bhutan (South Asia; “Bhutan”), Silverhull 24 from Canada (North America; “Silver Hull”), La Harpe from France (Western Europe), and Panda and Emka from Poland (Central Europe). Achenes of all genotypes, except Panda, were obtained from the Gene Bank of Crop Research Institute, Prague, Czech Republic, while achenes of Panda were obtained from the Gene Bank of the Slovak Republic, Piestany, Slovakia. The variety Emka is a tetraploid (4n = 32), while the other used genotypes are diploid (2n = 16).

The buckwheat fruits were surface-sterilized in a 0.3% potassium permanganate solution for 15 min, rinsed with distilled water, and placed on wet filter paper in Petri dishes for imbibition. The Petri dishes were then placed for 2 days in a dark growth chamber at 24 °C for 16 h and 22 °C for 8 h, with a relative humidity of 30%. The imbibed buckwheat fruits were transferred to small pots containing 30 g of fresh peat substrate (TS2 Klasmann-Deilmann, Geeste, Germany). The pots were placed in a growth chamber with 14 h of photoperiod, light intensity of 160 µM m^− 2^ s^− 1^, temperature of 25 °C and 22 °C during daytime and nighttime, respectively, and relative humidity of 60%. After 7 days, the buckwheat seedlings were transplanted into 3 L phenotyping pots filled with 920 g of fresh peat substrate (30 g from the small pot and an additional 890 g). 920 g of fresh substrate corresponds to 327 g of oven-dried substrate.

### Phenotyping platform setup and drought stress treatment

After the transplantation, the buckwheat plants were transferred to the Slovak PlantScreen™ Phenotyping Unit (SPPU) at the AgroBioTech research center of the Slovak University of Agriculture in Nitra, Slovakia. SPPU consists of the cultivation facility with controlled environmental conditions and the fluorescence camera (FLUORCAM), RGB, and hyperspectral imaging units (described below and depicted in Supplement S1).

A combination of white and red LED bars (Photon Systems Instruments, Drasov, Czech Republic) was used as a light source, while the transparent roof of the facility was covered with a black cloth [15]. Photoperiod was set to 16 h with 14 h of light with an intensity of 1100 µM m^− 2^ s^− 1,^ and the first and the last hour of the day gradual increase and decrease of the light intensity. The temperature was set to 25 °C (day) and 22 °C (night) with a relative humidity of 40%, but it varied due to the temperature control system’s inefficiency caused by a malfunction. The pattern of actual temperature and relative humidity is presented in Supplement S2.

The experimental design included 18 plants from each buckwheat genotype (Bhutan, Emka, La Harpe, Panda, Silver Hull, and Zimbabwe) randomly placed on the belts of the cultivation facility of SPPU. The position of the plants in the cultivation space was changed every second day to avoid the margin effect. All plants were watered to 80% of field water capacity (FWC) until the start of water stress treatment 22 days after sowing (DAS) when plants were at the beginning of flower buds development. From this moment on, plants were divided into three water treatment groups (with 6 plants in each) based on the actual quantum yield of photosystem II (PSII) measured from the top of whole plants on the previous day by the FLUORCAM imaging unit of SPPU. The plants of each variety were divided in the following way: From the lowest to the highest division followed the pattern: C, MS, SS, SS, MS, C, C, MS…, C, where C is the control (watering to 80% FWC), MS is Mild water Stress (watering to 50% FWC), and SS is Severe water Stress (watering to 30% FWC). As 100% FWC of the used substrate is 5.495 g.g^− 1^, the amount of water added to pots to reach 80% FWC was 1437.5 g. The watering of the MS and SS plants was disrupted at the beginning of the experiment’s main phase until they reached 50% or 30% of FWC, corresponding to 898.4 and 539.1 g of water, respectively. The irrigation was performed by the automated watering system of the SPPU every second day to reach the desired weight of the pot according to the water regime. The weight of pots was recorded before and after irrigation and was used to calculate evapotranspiration in g.day^− 1^. The weighting was always performed, including the plant mass.

### Automated hyperspectral and RGB imaging

The automated measurements by the hyperspectral unit of SPPU were performed on 1st, 5th, and 8th day after treatment (DAT). The measurements were taken using a Visible/Near-Infra-Red (VNIR) UltraSpec hyperspectral camera (PSI, Drasov, Czech Republic) of the hyperspectral imaging unit. The VNIR camera (silicon detector) covers the spectral range of 340–900 nm with an effective pixel resolution of 640 × 480 pixels, an effective pixel size of 25 μm, and a spectral resolution of 0.8 nm. Measured digital numbers were converted to surface reflectance using Spectralon panel readings and dark current correction. Pixels containing the plants were selected using values calculated according to Eq. 1 (ρ represents reflectance at a given wavelength) and threshold level > 0.2. The average reflectance of the whole plant was then used to calculate the vegetation indices (VIs) per the formulas described in Table 1.

1 $$\:1.2(2.5\left(\rho\:740-\rho\:672\right)-0.5\left(\rho\:740-\rho\:556\right))$$

**Table 1:** Formulas used to calculate Normalized Difference Vegetation Index (NDVI), Photochemical Reflectance Index (PRI), Vogelmann 2 index (Vog2), Anthocyanin Reflectance Index (ANTH), Carotenoid Reflectance Index (CAR); ρ – reflectance at the given wavelength

VI	Formula	Reference
NDVI	(ρ800-ρ670)/(ρ800+ρ670)	[16]
PRI	(ρ531-ρ570)/(ρ531+ρ570)	[17]
Vog2	(ρ734-ρ747)/(ρ715+ρ726)	[18]
ANTH	(1/ρ530- 1/ρ673)*ρ776	[19]
CAR	(1/ρ507- 1/ρ603-0.6456*1/ρ530)*ρ776	[19]

**Fig. 1:**
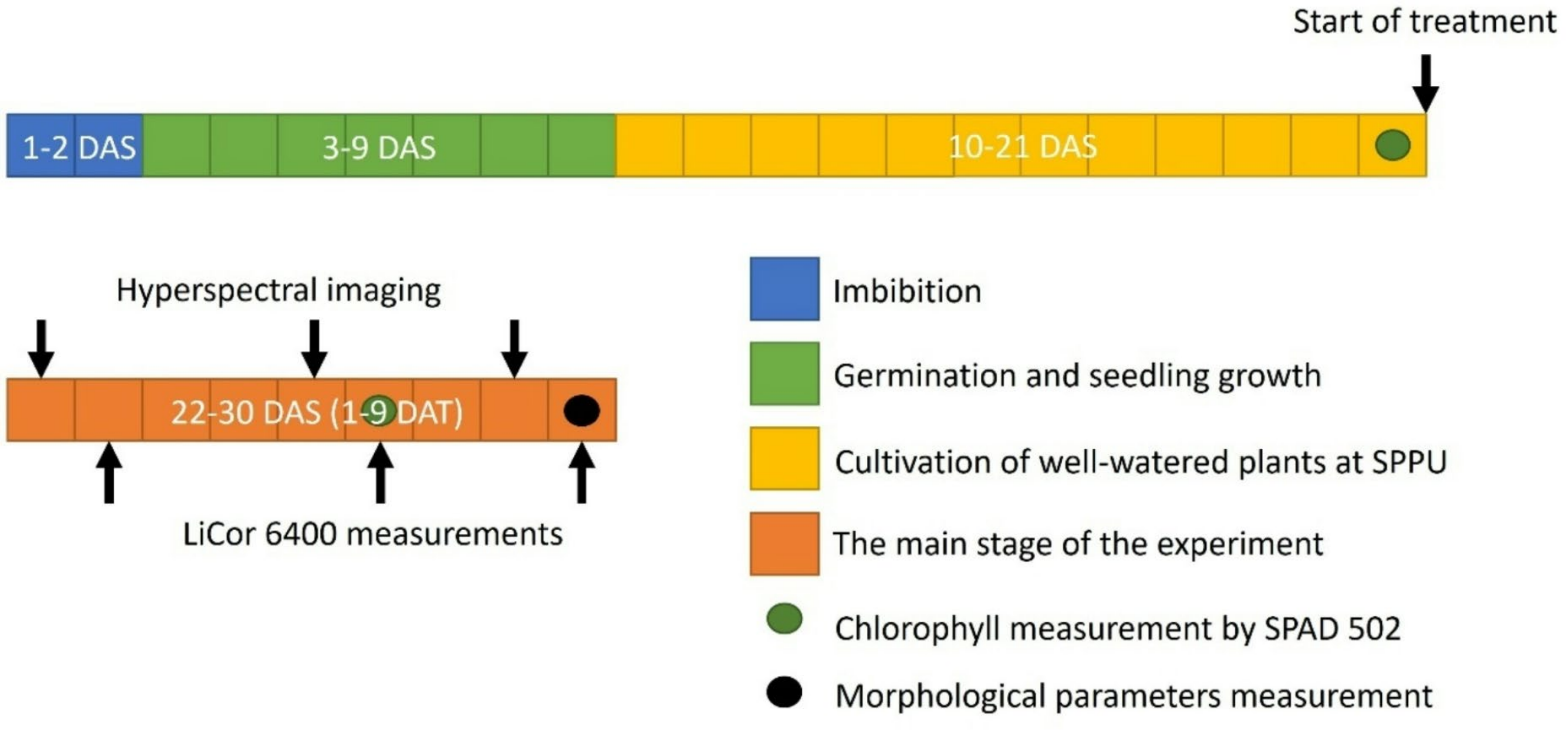
The timeline of the experiment and performed measurements. DAS and DAT stand for days after sowing and days after treatment, respectively

The RGB imaging unit, consisting of horizontal and 360°-turning vertical GigE uEye^®^ UI-5480SE-C – 5 Megapixel QSXGA camera with 1/2” CMOS sensor (Opto Engineering, Italy) and SV-0814 H lens (VS Technology, Japan) was used for the morphological parameters’ retrieval on 9th DAT (Fig. 1). The parameters were calculated using the previously obtained calibration curves to convert the pixel counts to mm. The top-view perimeter and height of plants were retrieved from the top-view and side-view images, respectively. The plant area (A^plant^) was calculated using the top-view area (A^top^) and two side-view images taken from 0° (A^side 0^) and 90° (A^side 90^) positions according to Eq. 2.

$$\:{A}^{plant}=\:\sqrt{{\left({A}^{top}\right)}^{2}+{\left({A}^{side\:0}\right)}^{2}+\:{\left({A}^{side\:90}\right)}^{2}\:}$$

The difference between plant area on the 21st DAS and 13th DAS and between the 30th DAS and 21st DAS was used to calculate the plant area growth in m^2^.day^− 1^ for the period before and after treatment initiation, respectively. Plant area growth and evapotranspiration for the abovementioned periods were subsequently used to calculate evapotranspiration per plant area growth in l.m− 2.

### Hand-held measurements

SPAD 502 (Konica Minolta Business Solutions, Europe) was used on the 21st DAS, a day before the treatment started, and on the 6th DAT to assess the chlorophyll content of the third leaf of every plant (Fig. 1).

The open infrared gas exchange system, Licor 6400 (LiCOR, USA), equipped with a fluorescence component, was used for gas exchange and chlorophyll fluorescence emission measurements on the 2nd, 6th, and 9th DAT (Fig. 1). To ensure consistency with the conditions in the growth room, the parameters within the measuring head were configured as follows: a leaf temperature of 25 °C, a reference CO_2_ concentration of 400 µL L^− 1^, and maintaining ambient air humidity within the range of 40–60%. The light source comprised a red LED light unit, complemented by an additional 10% blue LED light to aid in the opening of stomata. The assimilation measurements were performed with the light intensity of 1000 µM m^− 2^ s^− 1^ on the third oldest leaf of plants. The rapid gas exchange measurements taking 3 min were followed by chlorophyll fluorescence parameters measurement. Water use efficiency (WUE) was calculated as a ratio of assimilation [µM CO_2_ m^− 2^ s^− 1^] and transpiration [mM H_2_O m^− 2^ s^− 1^] retrieved from gas exchange measurements.

### Statistical analysis

The basic number of observations per genotype and treatment was 6; however, due to the damage caused by manipulation during measurements, some plants had to be excluded. The number of observations included in the analysis is reported in the Supplement S3. Statistical significance was assessed through unbalanced type II two-way analysis of variance (ANOVA) for each measurement day individually with genotype and water regime as factors. Function *emmeans* from the “emmeans” package was used for performing the mentioned ANOVA. All statistical analysis was conducted using RStudio version 2022.02.0 + 443 (RStudio Inc., Boston, Massachusetts, USA).

## Results

### Effect of water stress on morphological parameters

**Fig. 2:**
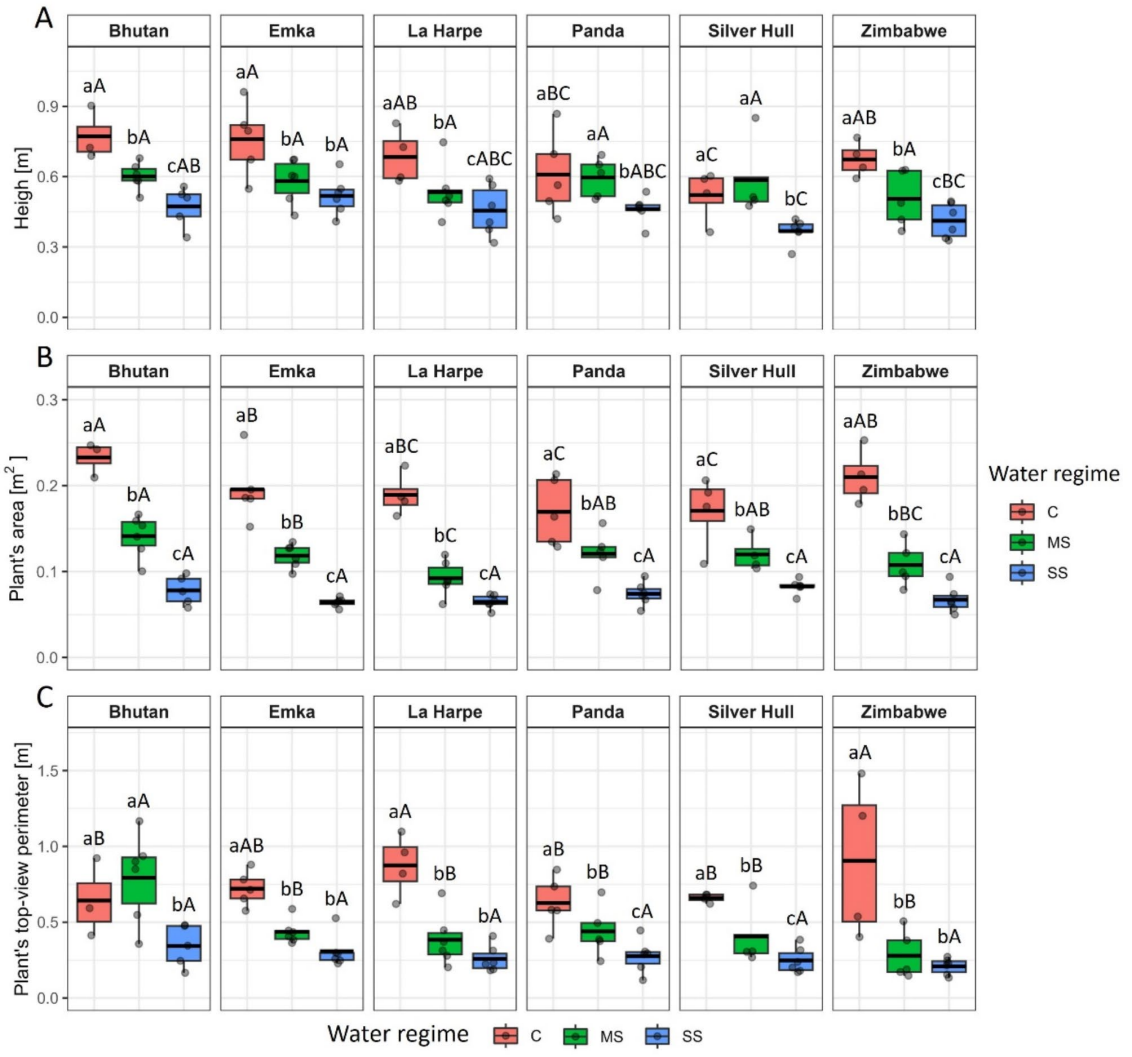
The impact of water stress on plant **A**) height, **B**) area, and **C**) top view perimeter in different buckwheat genotypes under control (C), mild stress (MS) and severe stress (SS) conditions 9 DAT (30 DAS). The thick line in the boxplot represents the mean. Different lowercase letters represent statistically significant differences among different water regimes for a given genotype, and different uppercase letters represent statistically significant differences among genotypes for a given water regime (*p* < 0.05)

The buckwheat genotypes were studied for their variation in morphological parameters such as plant height, plant area, and plant top-view perimeter 30 DAS (9 DAT). Moreover, the plant growth expressed as the increment of the plant area was determined for the period before the start of treatment (13–21 DAS) and after the start of treatment (21–30 DAS; Supplement S4). A genotype-dependent effect of drought was observed on plant height. While water stress caused gradual shortening of plants with increasing severity for Bhutan, La Harpe, and Zimbabwe, it had no significant effect on Panda and Silver Hull plants under MS. However, SS decreased the plant height of all genotypes in comparison to C. SS had no different impact than MS on height of Emka. All genotypes had a similar height under MS conditions, while under SS, Silver Hull was the shortest and Bhutan with Emka the tallest and similar to C conditions (Fig. 2A).

The plant area gradually decreased with the water stress severity for all studied genotypes (Fig. 2B). However, the magnitude of the plant area decrease among genotypes was different. MS caused 51%, 49%, 39%, 39%, 30% and 29% decrease in area for La Harpe, Zimbabwe, Bhutan, Emka, Silver Hull and Panda, respectively. The plant area under SS was, on average, smaller by 68% for Zimbabwe, 67% for Bhutan and Emka, 66% for La Harpe, 56% for Panda and 52% for Silver Hull compared to the respective C. In C conditions, Bhutan and Zimbabwe plants grew significantly larger than Panda and Silver Hull, with Bhutan being also larger than Emka and La Harpe. The plant area 30 DAS shows the same pattern as the growth after the drought stress initiation (Supplement S4B), while Supplement S4 A suggests that the changes in plant morphology were indeed caused by the water regime and not by inherited differences in the growth of plants.

The plant’s top-view perimeter, which indicates the plant’s branch growth, decreased due to MS and SS in all genotypes except Bhutan, in which case only SS caused a significant decrease (Fig. 2C). While in C, the plants with the largest perimeter were of Zimbabwe and La Harpe genotypes, in MS, Bhutan plants had the largest perimeter.

## Effect of water stress on vegetation indices

**Fig. 3:**
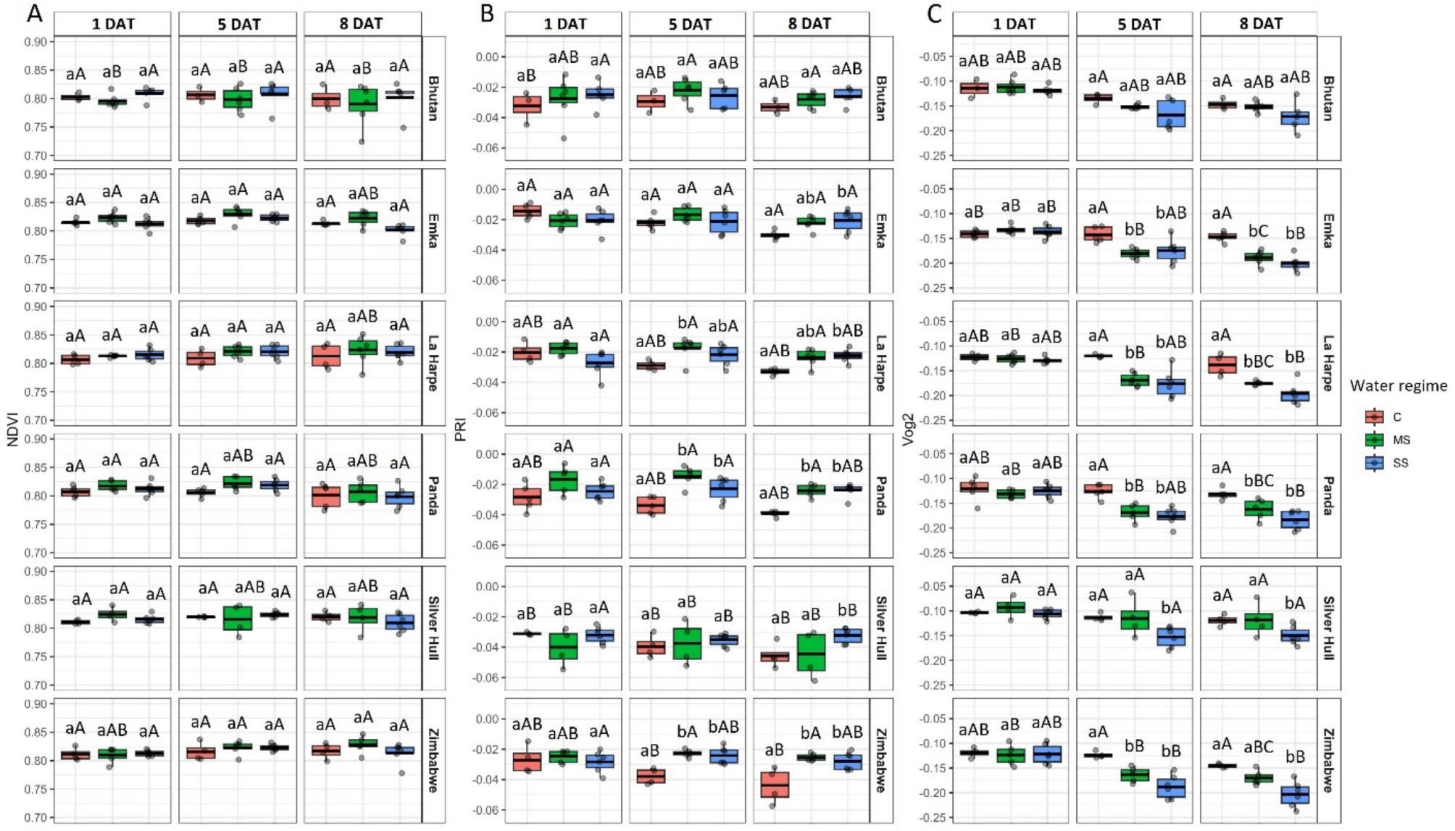
The impact of water stress on **A**) Normalized Difference Vegetation Index (NDVI), **B**) Photochemical Reflectance Index (PRI) and **C**) Vogmann 2 index (Vog2) in different buckwheat genotypes (in rows) under control (C), mild stress (MS) and severe stress (SS) conditions 1, 5, and 8 days after treatment (DAT; in columns). The thick line in the boxplot represents the mean. Different lowercase letters represent statistically significant differences among different water regimes for a given genotype and day, and different uppercase letters represent statistically significant differences among genotypes for a given water regime and day (*p* < 0.05)

Our observations indicate no statistically significant differences in the Normalized Difference Vegetation Index (NDVI) between C and stressed plants (Fig. 3A). However, the Photochemical Reflectance Index (PRI) showed a significant increase for stressed plants of Panda and Zimbabwe as early as 5 DAT. Moreover, 8 DAT, a significant increase of PRI was observed for MS plants of Panda and Zimbabwe as well as SS plants of all genotypes except Bhutan (Fig. 3B).

A gradual decrease of Vogelmann 2 (Vog2) index with increasing water stress severity, with a significant difference between C and SS plants, was observed for Bhutan 5 and 8 DAT. Both MS and SS caused significantly lower Vog2 of Emka, La Harpe, and Panda plants on the 5th and 8th DAT. Although MS and SS caused a significant decrease in Zimbabwe plants Vog2 5 DAT, 8 DAT, only SS plants showed significantly lower Vog2 compared to C. The lowest impact of water stress on Vog2 was observed in the case of the Silver Hull genotype when only SS on the 5th DAT caused a significant decrease of Vog2 compared to the respective C (Fig. 3C).

**Fig. 4:**
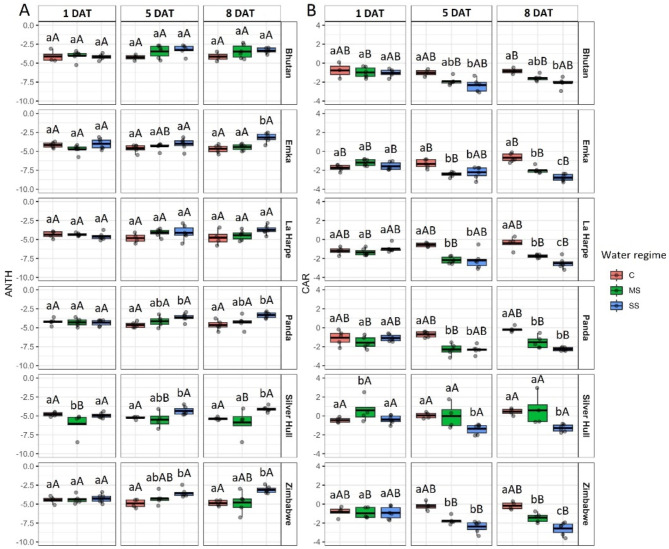
The impact of water stress on **A**) Anthocyanin Reflectance Index (ANTH) and **B**) Carotenoids Reflectance Index (CAR) in different buckwheat genotypes (in rows) under control (C), mild stress (MS) and severe stress (SS) conditions 1, 5, and 8 days after treatment (DAT; in columns). The thick line in the boxplot represents the mean. Different lowercase letters represent statistically significant differences among different water regimes for a given genotype and day, and different uppercase letters represent statistically significant differences among genotypes for a given water regime and day (*p* < 0.05)

The observations of the Anthocyanin Reflectance Index (ANTH) showed an increase in its value in SS plants on the 5th DAT for Panda and Zimbabwe genotypes, while on the 8th DAT, significantly higher values were observed also for the Emka genotype (Fig. 4A). The Carotenoid Reflectance Index (CAR) shows a significant decrease in its value 5 DAT for MS and SS plants of all genotypes except Silver Hull, in which case only SS caused significantly lower values. The gradual significant reduction of CAR values with increasing severity of water stress on 8th DAT was observed for Emka, La Harpe, Panda, and Zimbabwe genotypes, while only SS was found to significantly reduce CAR on that day for Bhutan and Silver Hull (Fig. 4B).

**Fig. 5:**
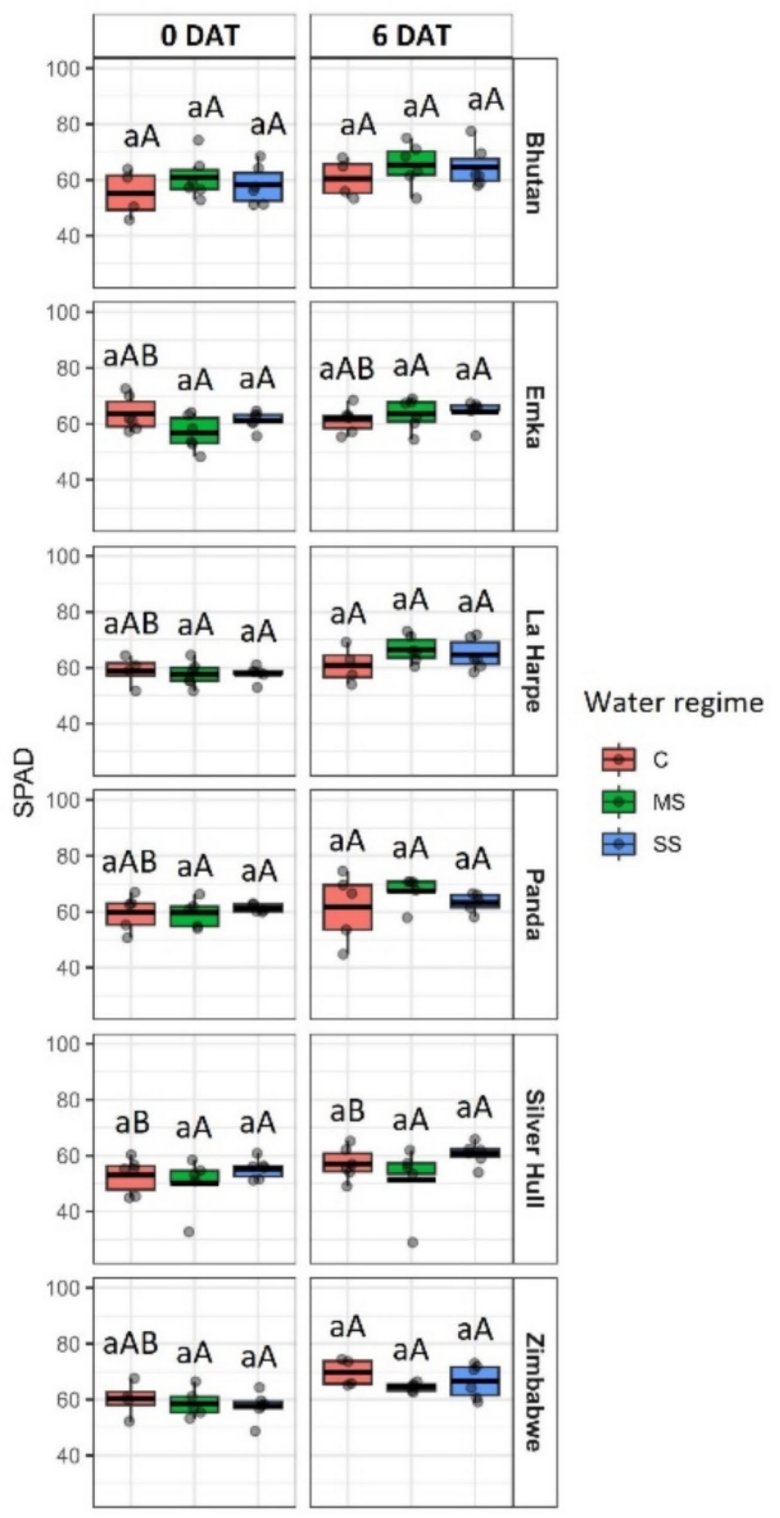
The impact of water stress on chlorophyll content (in SPAD unit) in different buckwheat genotypes (in rows) under control (C), mild stress (MS) and severe stress (SS) conditions before the start of the treatment and 0 days after treatment (DAT; in columns). The thick line in the boxplot represents the mean. Different lowercase letters represent statistically significant differences among different water regimes for a given genotype and day, and different uppercase letters represent statistically significant differences among genotypes for a given water regime and day (*p* < 0.05)

Relative chlorophyll content measured in the SPAD unit suggests no significant changes in chlorophyll concentration 6 DAT (Fig. 5).

### Effect of water stress on selected plant physiological processes

**Fig. 6:**
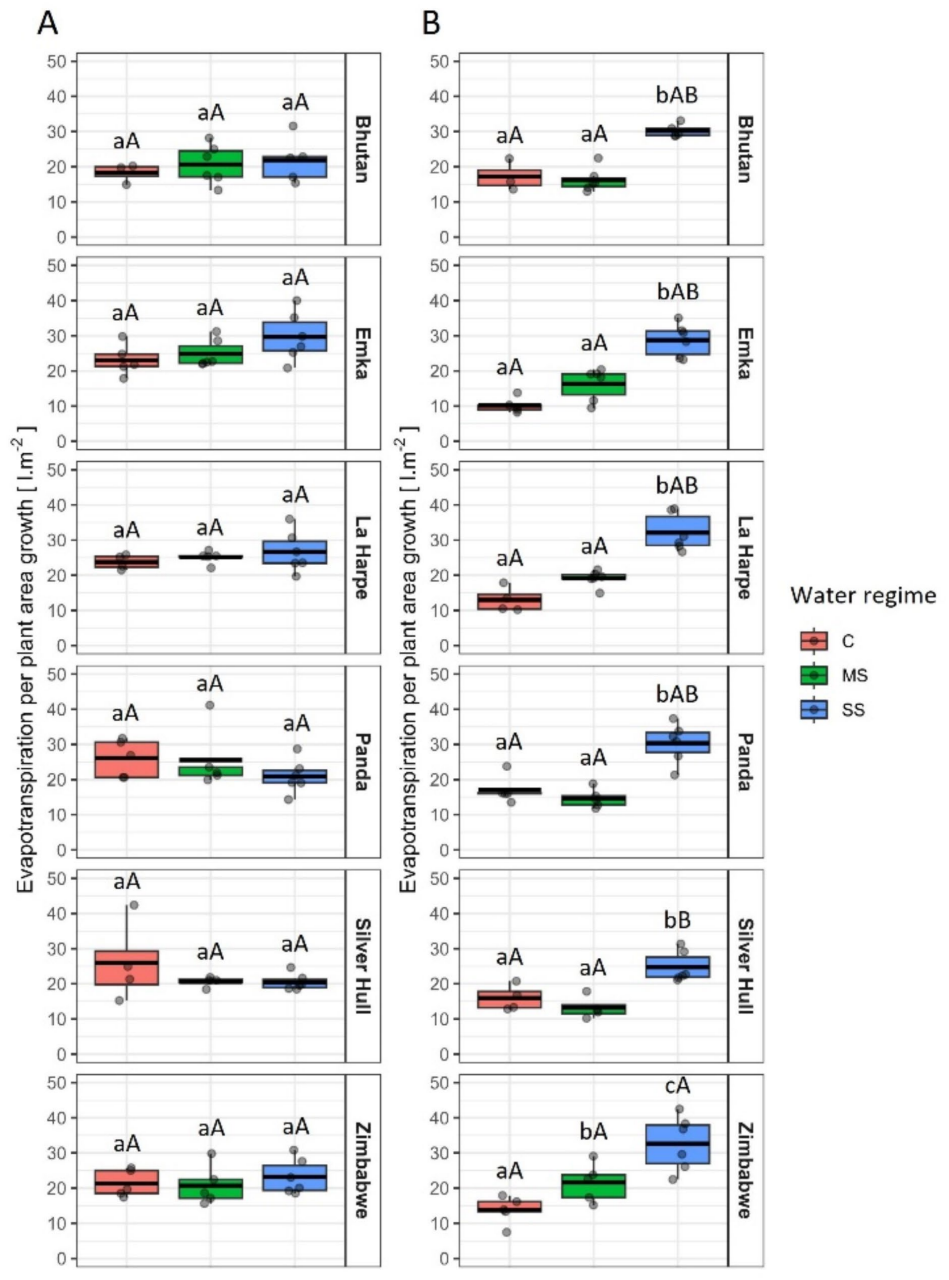
The evapotranspired water amount for a meter square of plant area production **A**) before the start of the treatment (13–21 DAS), and **B**) after the start of treatment (21–30 DAS) in different buckwheat genotypes under control (C), mild stress (MS) and severe stress (SS). The thick line in the boxplot represents the mean. Different lowercase letters represent statistically significant differences among different water regimes for a given genotype and day, and different uppercase letters represent statistically significant differences among genotypes for a given water regime and day (*p* < 0.05)

Plant area growth and the evapotranspiration of plants of all genotypes were uniform before the start of treatment (Supplement S4 A, S5 A). As a result, before the start of the treatment, all plants, irrespective of genotype, exhibited similar levels of evapotranspiration per plant area growth. However, in the period from the start of treatment until 9 DAT, SS caused significantly lower growth of plant area per water evapotranspired for all genotypes, and MS lowered this growth in the case of Zimbabwe (Fig. 6). These differences were caused by a disproportionally larger decrease in plant area growth compared to evapotranspiration of plants grown under SS in comparison to C (Supplement S4B, S5B).

**Fig. 7:**
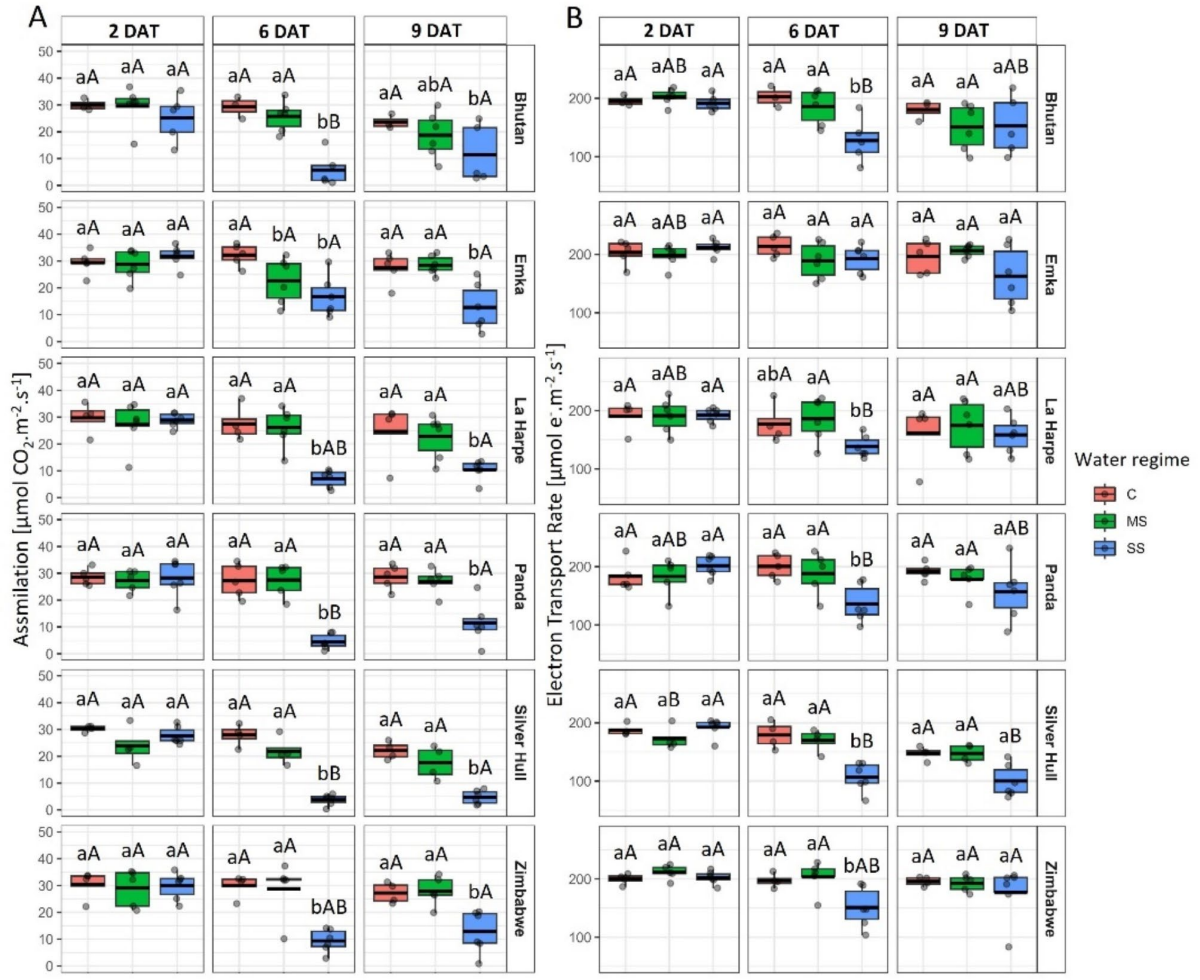
The impact of water stress on **A**) CO_2_ assimilation rate, and **B**) Electron transport rate in different buckwheat genotypes (in rows) under control (C), mild stress (MS) and severe stress (SS) conditions 2, 6, and 9 days after treatment (DAT; in columns). The thick line in the boxplot represents the mean. Different lowercase letters represent statistically significant differences among different water regimes for a given genotype and day, and different uppercase letters represent statistically significant differences among genotypes for a given water regime and day (*p* < 0.05)

CO_2_ assimilation rate significantly decreased in all the studied genotypes under SS conditions 6 and 9 DAT compared to C (Fig. 7A). On the 6th DAT, a significantly reduced electron transport rate (ETR) of SS plants compared to C plants was observed in all genotypes except Emka. However, 9 DAT, the significant reduction was only observed in genotype Silver Hull under SS compared to C (Fig. 7B).

**Fig. 8:**
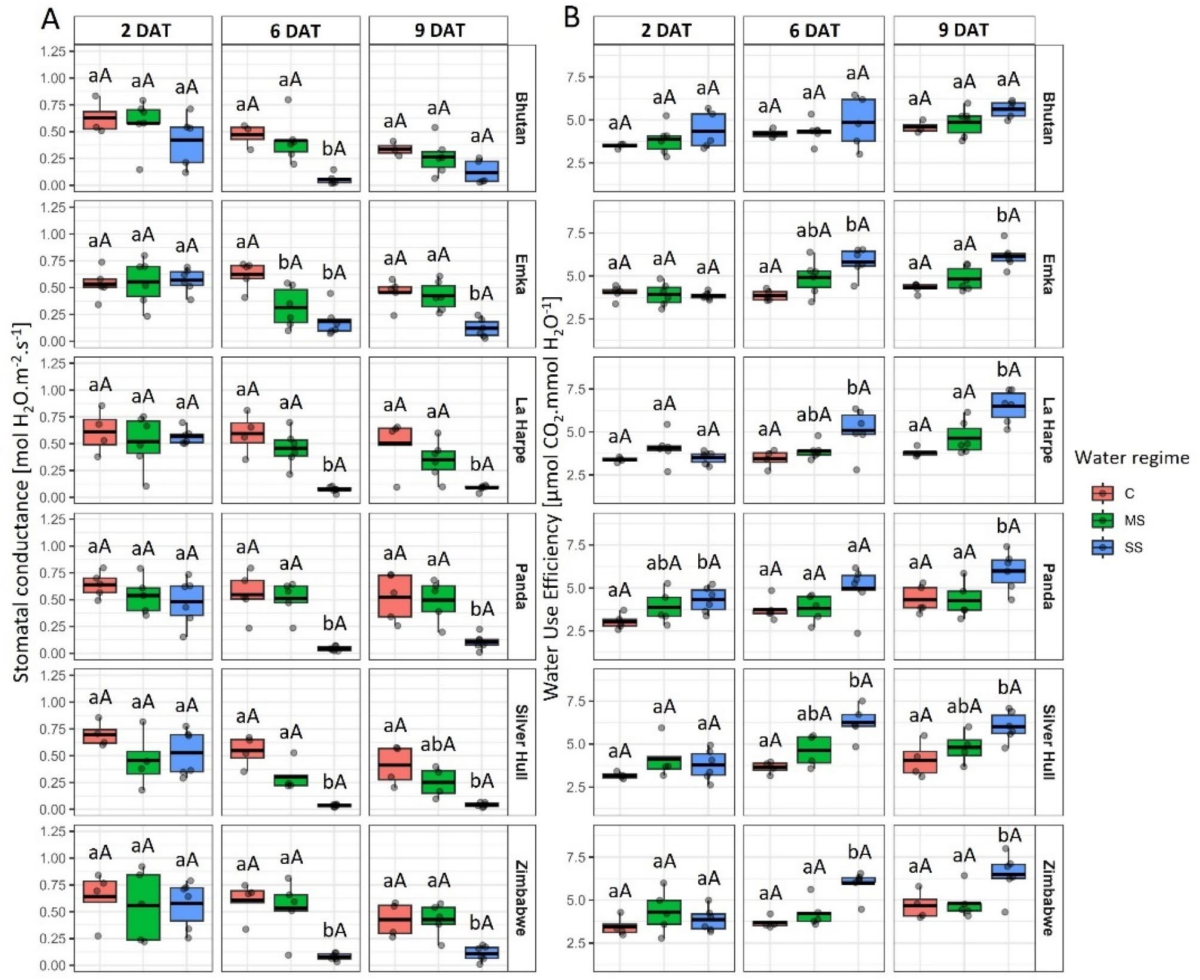
The impact of water stress on **A**) Water Use Efficiency (WUE) and **B**) Stomatal Conductance (g_s_) in different buckwheat genotypes (in rows) under control (C), mild stress (MS), and severe stress (SS) conditions 2, 6, and 9 days after treatment (DAT; in columns). The thick line in the boxplot represents the mean. Different lowercase letters represent statistically significant differences among different water regimes for a given genotype and day, and different uppercase letters represent statistically significant differences among genotypes for a given water regime and day (*p* < 0.05)

The stomatal conductance (*g*_*s*_) significantly decreased, whereas WUE significantly increased for all the studied buckwheat genotypes under SS conditions 6 and 9 DAT. MS caused decreased g_s_ and increased WUE in the case of the Emka genotype on the 6th DAT and increased WUE for Bhutan plants 9 DAT (Fig. 8).

## Discussion

Buckwheat is considered to be a drought-resistant crop, which adjusts its physiological processes as per the water availability [10]. Given the forthcoming threat of climate change and the increasing likelihood of more frequent drought events [8], there is a need to constantly seek alternative crops that can better suit the environment. In this study, the automated phenotyping unit was employed to collect a relatively large dataset to analyze the six genotypes of buckwheat subjected to MS and SS conditions. Despite originating on different continents in different climatic regions, the genotypes showed surprisingly similar growth responses to the drought. The observed decrease in morphological parameters such as plant height, area, and top-view perimeter under severe water stress aligns with previous research [10, 20]. Combined with the decreased growth rates, these data indicate the resource allocation shift toward survival strategies in response to water scarcity. The smaller plants under drought conditions are generally linked to a decrease in cell expansion, impaired mitosis, and an increase in leaf shedding [21]. In studied buckwheat genotypes, we did not observe any shedding under MS or SS conditions, showing its resistant nature. Among six studied buckwheat genotypes, Panda and Silver Hull maintained their height under MS, whereas the plant area for these varieties under MS decreased the least in comparison to respective Cs. As Panda and Sliver Hull were observed to be the shortest and smallest genotypes by plant area in C conditions, our results show the advantage of the shorter genotypes in coping with MS.

The results regarding vegetation indices (NDVI, PRI, and Vog2) and biochemical markers (ANTH and CAR) highlight the complexity of assessing drought stress in buckwheat. The Vog2 index shows clear differences between control and water stress-exposed plants (Fig. 3C). Vog2 was developed to observe the changes in the red-edge spectrum of vegetation that occur due to the combined effects of chlorophyll content and leaf structure alterations [18, 22]. Leaf moisture content, along with other plant stress-causing factors, impact the shape of the red edge reflectance [23]. Previously, red edge-related vegetation indices have been observed to identify drought stress in different plants [23, 24]. In our study, Vog2 appears to be a reliable indicator of drought stress, potentially reflecting the buckwheat’s ability to adapt and respond to changing conditions. The NDVI is generally regarded as a proxy of plant biomass and chlorophyll content [25]. Because in our study, we used only pixels containing plants, we reduced the information contained in NDVI values only to chlorophyll content. As chlorophyll content did not vary under different water regimes (Fig. 5), no significant differences in NDVI values due to limited water supply were observed (Fig. 3A). The CAR index was developed as a measure of carotenoid content when the decreasing value of the index is interpreted as increasing the content of carotenoids [19]. Therefore, our results indicate a higher accumulation of carotenoids under drought stress in buckwheat genotypes (Fig. 4). PRI is another index sensitive not only to the carotenoids content of the plants but also to their qualitative profile [17]. In our study, we observed a simultaneous significant decrease in CAR and an increase in PRI, only for Emka, La Harpe, Panda, and Zimbabwe under SS (Figs. 3 and 4). However, a significant decrease in carbon assimilation (Fig. 7) and plant growth (Fig. 2) was better reflected in the results of CAR (Fig. 4B). Moreover, the change in the CAR index occurred as soon as 5 DAT, indicating its good performance in detecting a reaction to water limitation for buckwheat genotypes. The concentration of anthocyanins is reflected in the ANTH index when a higher index value indicates a higher concentration [19]. ANTH and CAR indices provide insights into the specific adaptive strategies employed by different genotypes. All genotypes showed increased carotenoids level as a protective mechanism, while only Emka, Panda, Silver Hull, and Zimbabwe exhibited a significantly increased anthocyanins level, possibly indicating their reliance on other mechanisms for coping with drought. The observation of anthocyanins and carotenoids increase is in agreement with previous studies focused on plant response under drought stress [26, 27].

The observations of chlorophyll content and photosynthetic properties, such as CO_2_ assimilation rate and ETR, suggest that different buckwheat genotypes employ similar strategies to cope with drought. Different studies have shown a decrease or increase in chlorophyll content under drought [21, 28, 29]), whereas, in this study, no significant change in chlorophyll content in 6 DAT was observed. A decrease in chlorophyll content is generally related to long-term severe drought stress, but an early stress such as 6 DAT appears not to impact chlorophyll synthesis in relation to plant growth, and the ratio of plant growth to chlorophyll remains the same resulting in similar chlorophyll content per unit of leaf area. Even if there was no decrease in chlorophyll in 6 DAT, a significant decrease in carbon assimilation was observed in both MS and SS conditions. This decrease can be attributed to stomatal closure as a reaction to limited soil water availability (Fig. 8A) [30]. Under conditions of decreased CO_2_ assimilation and unchanged light intensity, the harvested energy has to be dissipated in different way to avoid damage to the photosynthetic apparatus [31, 32]. In all genotypes, except Silver Hull, ETR decreased 6 DAT under SS condition and recovered to the same level as the control in 9 DAT (Fig. 7B). This suggests the acclimation of the electron transport chain (ETC) to drought in those genotypes. The lower magnitude of changes in ETR in comparison to CO_2_ assimilation clearly indicated that harvested energy was used differently than for carbon assimilation, as indicated by others [33, 34]. As a recovery of ETR did not result in a recovery of CO_2_ assimilation rate due to stomatal limitation, the accumulation of photochemical energy may result in redox poise on ETC [35]. Carotenoids and anthocyanin act as light-harvesting pigments, as well as antioxidants, that can protect chlorophyll and membrane destruction by quenching free radicle molecules from the leaf [36, 37]. Therefore, the abovementioned increase in photoprotective pigments such as anthocyanins and carotenoids, together with changes in the xanthophyll cycle, indicate a protective mechanism adopted by buckwheat to protect electron transport chain from redox poise under drought conditions.

WUE and *g*_*s*_ exhibited significant changes under severe water stress, underscoring the physiological adaptations of buckwheat to conserve water under drought conditions (Fig. 8). The increased WUE and decreased *g*_*s*_ indicate that the plants are optimizing their water use in response to the reduced availability of this essential resource, as reported by [30]. The decline in *g*_*s*_ can be associated with plant age and decreasing leaf area and mass under drought conditions [38]. The buckwheat genotypes have shown survival strategies by sensing the water stress at an early stage, minimizing the *g*_*s*_ and increasing its WUE, which is visible even in mild water stress conditions. However, the increased WUE at the leaf level was not translated to higher plant area growth per evapotranspiration. On the contrary, evapotranspiration per plant area growth of plants under SS increased (Fig. 6). This seemingly opposite information about water use in drought stress conditions probably stems from the reallocation of assimilated carbon from growth into secondary metabolite production, as discussed in connection to protective pigments and light energy use. Such high plasticity in altering between primary and secondary metabolism is probably the main reason for the high buckwheat drought resistance reported before [9, 39]. Our results support the previous findings that buckwheat is a good candidate for a wheat alternative under drought conditions [39].

## Conclusions

The results obtained by the automated phenotyping platform, along with traditional leaf-level measurements, collectively illustrate the intricate web of responses that buckwheat genotypes employ to mitigate the impact of water stress. The morphological changes are interconnected with physiological and biochemical adaptations, and the differential responses in changes of morphology and protective pigment accumulation observed among genotypes highlight the role of genetic diversity in implementing unique strategies for drought resistance. These results suggest that buckwheat’s response to water stress involves a combination of morphological, physiological, and biochemical adjustments, which vary on the genotype level and depend on the severity of the stress. Out of the studied genotypes, Panda proved to be the most drought resistant as its height and plant area were the least affected by MS; it rapidly responded to drought by higher protective pigments accumulation, and its ETR recovered even under SS. Silver Hull was another genotype that could maintain its aboveground biomass under MS at a similar level as C. These results are contrary to our hypothesis as these two genotypes originate in cold countries (Poland and Canada), and the genotype Zimbabwe from Africa was found to be the most drought-sensitive. The magnitude of the response to drought was related to the biomass production potential, which was higher for the genotypes of warmer regions of origin (Bhutan, Zimbabwe) and lower for the cold regions of origin. Further research employing molecular analysis is needed to elucidate the underlying mechanisms driving these responses and enable faster development of drought-resistant buckwheat varieties.

## Electronic supplementary material

Below is the link to the electronic supplementary material.

Supplementary Material 1

## Data Availability

The raw data related to article has been deposited in the Open Data Repository RepOD (https://repod.icm.edu.pl/) – the PULS institutional repository with DOI: 10.18150/PQ3QGG.
