# Correction: High-throughput phenotyping of buckwheat (*Fagopyrum esculentum* Moench.) genotypes under water stress: exploring drought resistance for sustainable agriculture

**DOI:** 10.1186/s12870-025-06560-4

**Published:** 2025-04-24

**Authors:** Michal Antala, Marek Kovar, Lucia Sporinová, Andrej Filacek, Radosław Juszczak, Marek Zivcak, Aida Shomali, Raghavendra Prasad, Marian Brestic, Anshu Rastogi

**Affiliations:** 1 Laboratory of Bioclimatology, Department of Ecology and Environmental Protection, Faculty of Environmental Engineering and Mechanical Engineering, Poznan University of Life Sciences, Piątkowska 94, Poznań, 60-649 Poland; 2 Department of Plant Physiology, Faculty of Agrobiology and Food Resources, Slovak University of Agriculture, A. Hlinku 2, Nitra, 949 76 Slovak Republic; 3 Department of Botany and Genetics, Faculty of Natural Sciences and Informatics, Constantine the Philosopher University in Nitra, Nábrežie mládeže 573, Nitra, 949 01 Slovak Republic; 4 Department of Environmental Horticulture, Royal Horticultural Society, London, GU23 6QB UK; 5 Department of Botany and Plant Physiology, Faculty of Agrobiology, Food, and Natural Resources, Czech University of Life Sciences Prague, Kamycka 129, Prague, 165 00 Czech Republic

**Correction:** ***BMC Plant Biol*** **25, 444 (2025)**

10.1186/s12870-025-06429-6

Following publication of the original article [1], the authors found a minor typographical error in the auhor name of Ragh**a**vendra Prasad. The change is highlighted below in bold.

**The incorrect name is: **Raghvendra Prasad

**The correct name is: **Ragh**a**vendra Prasad

The correction does not compromise the validity of the conclusions and the overall content of the article. The author group has been updated above and the original article [1] has been corrected.
