# Supplementary material for: High-throughput phenotyping of buckwheat (Fagopyrum esculentum Moench.) genotypes under water stress: exploring drought resistance for sustainable agriculture
Source: BMC Plant Biol. 2025 Apr 8;25:444. doi: 10.1186/s12870-025-06429-6 (PMC11978128; doi:10.1186/s12870-025-06429-6)
Supplement: Supplementary file 1 — Supplementary Material 1 [file 12870_2025_6429_MOESM1_ESM.docx]

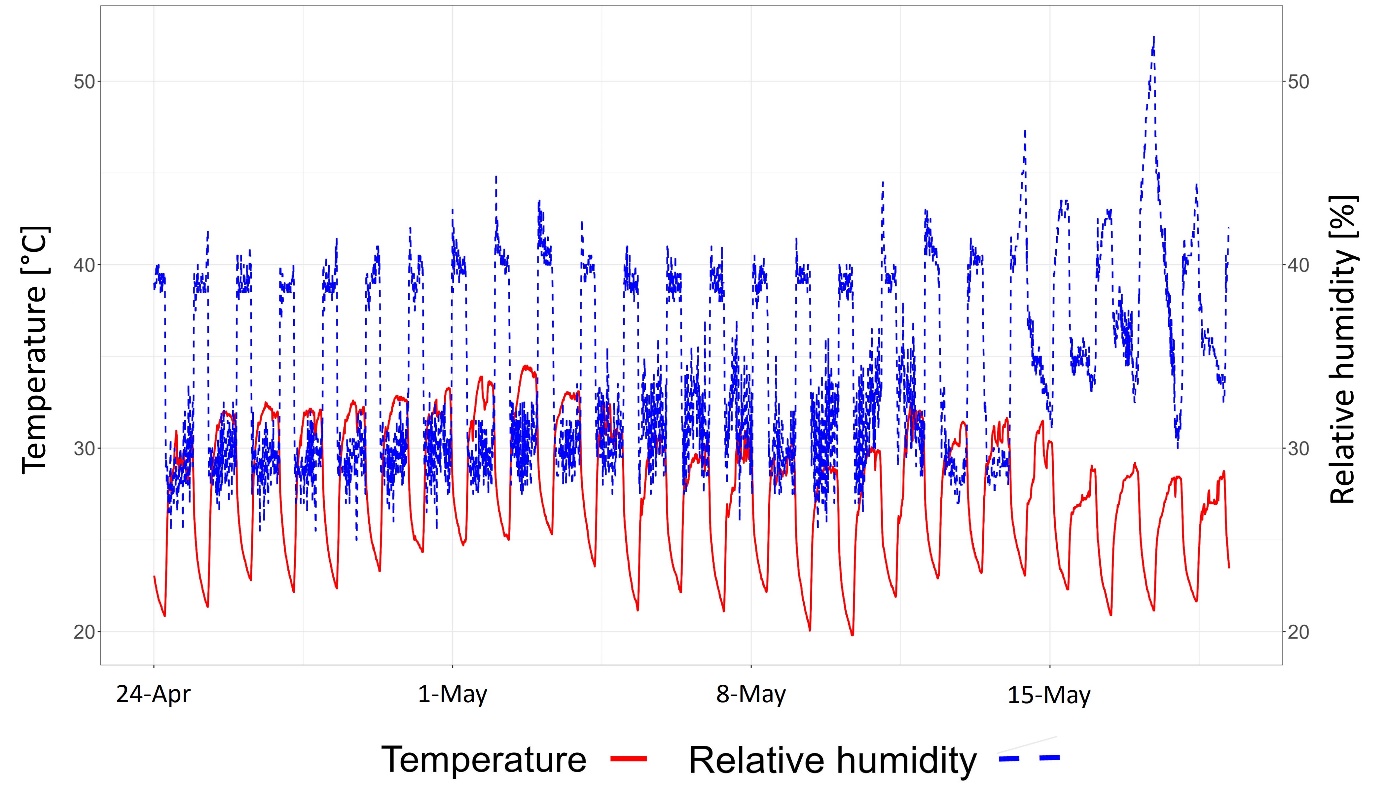
**
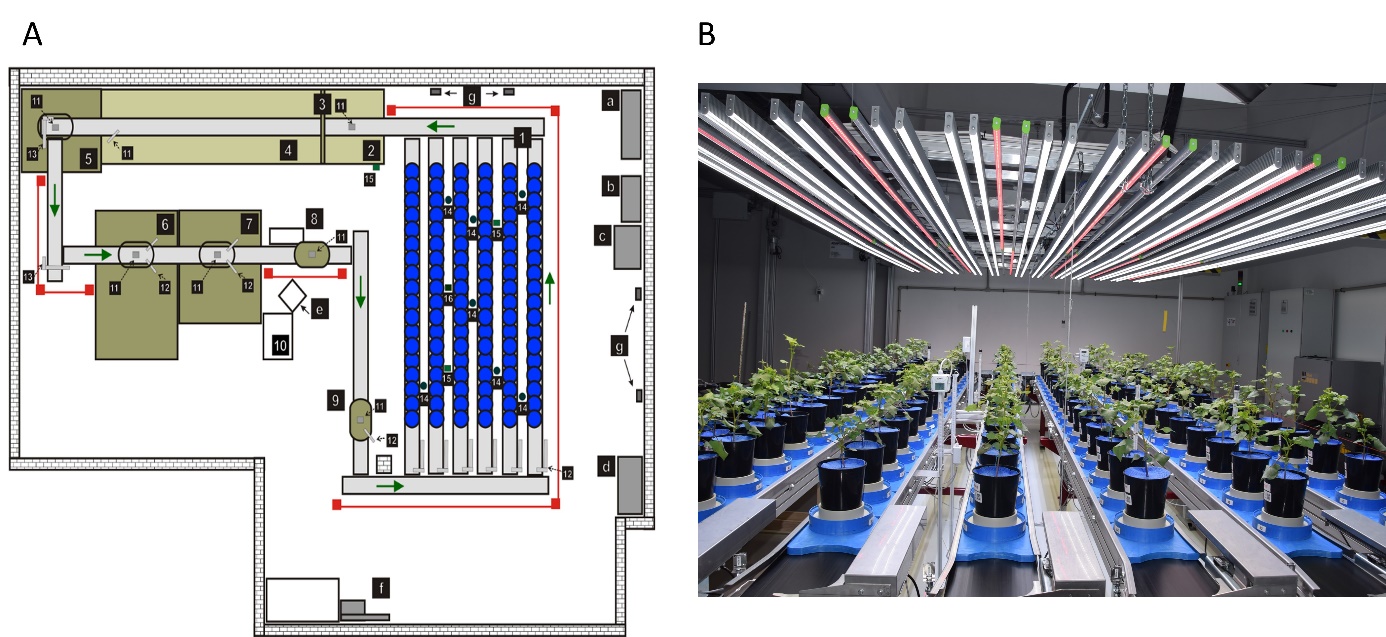
Supplement 1.** Schematic representation of Slovak PlantScreen^TM^ Phenotyping Unit (**A**) and the depiction of experimental plants placed in the cultivation facility of the unit (**B**). The numbers and letters in panel A represent: 1 – cultivation facility with LED light system, 2 – pre-adaptation unit, 3 – laser scanning gate, 4 – adaptation unit with LED system, 5 – FLUORCAM imaging unit, 6 – RGB imaging unit, 7 – hyperspectral imaging unit, 8 – weight and watering station, 9 – manual loading station, 10 – control PC and database box, 11 – tag reader, 12 – stopper, 13 – disc puller, 14 – PAR sensor, 15 – thermohygrometer, 16 – CO_2_ sensor, a – FLIER system cabinet, b – PLC cabinet, c – air compressor, d – LED light control cabinet, e- HMI panel, f- aqua osmotic system, g – ultrasonic humidifier.

**Supplement 2.** Temperature [°C] and relative humidity [%] in the cultivation facility of the Slovak PlantScreen^TM^ Phenotyping Unit during the experiment.

**Supplement 3.** The number of observations per genotype and treatment used for analysis. C – control, MS – Mild water Stress, SS – Severe water Stress

| **Genotype** | **C** | **MS** | **SS** |
| --- | --- | --- | --- |
| **Bhutan** | 3 | 6 | 5 |
| **Emka** | 5 | 6 | 5 |
| **La Harpe** | 4 | 6 | 6 |
| **Panda** | 5 | 5 | 6 |
| **Silverhull** | 5 | 4 | 6 |
| **Zimbabwe** | 4 | 5 | 6 |

**
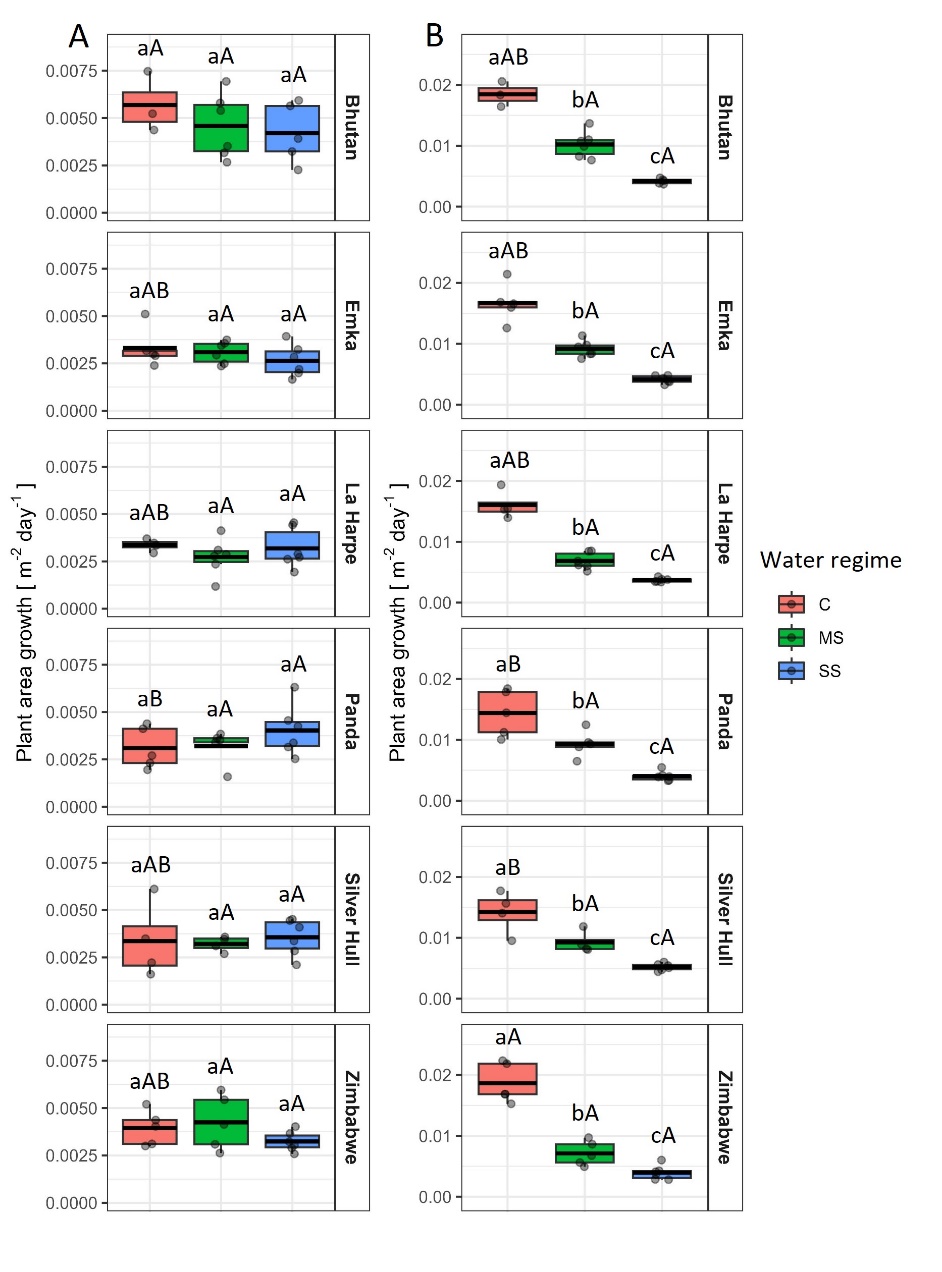
**

**Supplement 4.** The plant area growth **A**) before the start of the treatment (13-21 DAS), and **B**) after the start of treatment (21-30 DAS) in different buckwheat genotypes under control (C), mild stress (MS) and severe stress (SS). The thick line in the boxplot represents the mean. Different lowercase letters represent statistically significant differences among different water regimes for a given genotype and day, and different uppercase letters represent statistically significant differences among genotypes for a given water regime and day (p<0.05). Note that the y-axis **
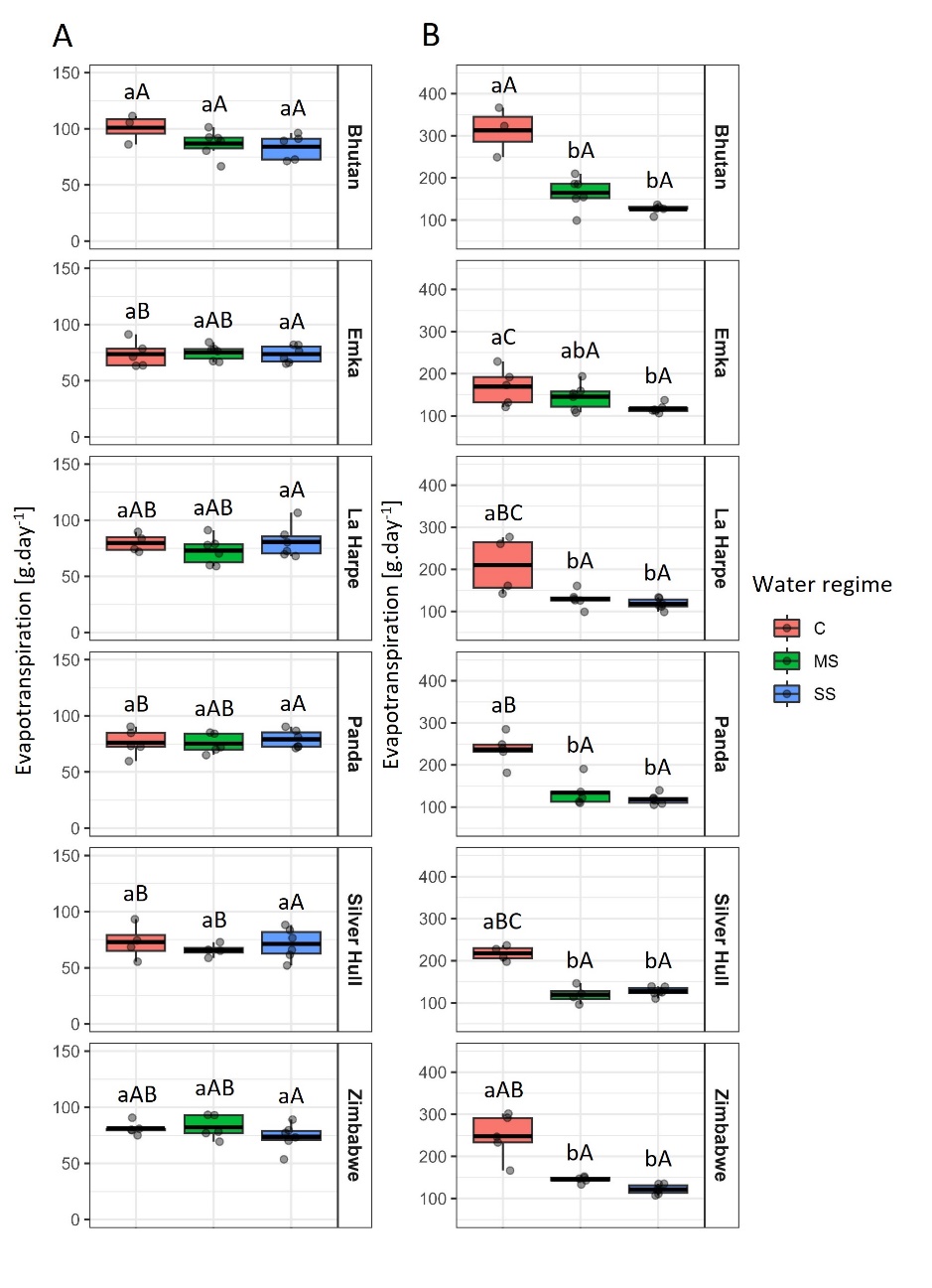
**for panels A and B are different due to presentation clarity.

**Supplement 5.** The pot evapotranspiration **A**) before the start of the treatment (13-21 DAS), and **B**) after the start of treatment (21-30 DAS) in different buckwheat genotypes under control (C), mild stress (MS) and severe stress (SS). The thick line in the boxplot represents the mean. Different lowercase letters represent statistically significant differences among different water regimes for a given genotype and day, and different uppercase letters represent statistically significant differences among genotypes for a given water regime and day (p<0.05). Note that the y-axis for panels A and B are different due to presentation clarity.
